# Superiority of integrated cervicothoracic immobilization in the setup of lung cancer patients treated with supraclavicular station irradiation

**DOI:** 10.3389/fonc.2023.1135879

**Published:** 2023-03-20

**Authors:** Bao Wan, Shihong Luo, Xin Feng, Wenhua Qin, Haifan Sun, Lu Hou, Kun Zhang, Shiyu Wu, Zongmei Zhou, Zefen Xiao, Dongfu Chen, Qinfu Feng, Xin Wang, Fukui Huan, Nan Bi, Jianyang Wang

**Affiliations:** Department of Radiation Oncology, National Cancer Center/National Clinical Research Center for Cancer/Cancer Hospital, Chinese Academy of Medical Sciences and Peking Union Medical College, Beijing, China

**Keywords:** lung cancer, radiotherapy, positioning error, thoracoabdominal flat immobilization device, integrated cervicothoracic immobilization device

## Abstract

**Objective:**

To investigate the superiority of the integrated cervicothoracic immobilization devices (ICTID) on the mobility of the supraclavicular station in lung cancer patients requiring both primary lung lesion and positive supraclavicular lymph nodes irradiation.

**Methods:**

One hundred patients with lung cancer were prospectively enrolled in the study. The following four different fixation methods are used for CT simulation positioning: thoracoabdominal flat immobilization device fixation with arms lifting (TAFID group), head-neck-shoulder immobilization device fixation with arms on the body sides (HNSID group), ICTID fixation with arms on the body sides (ICTID arms-down group), and n ICTID fixation with arms lifting (ICTID arms-up group). Cone-beam computed tomography (CBCT) images are taken daily or weekly before treatment, to assess anatomical changes during the radiotherapy course.

**Results:**

The translation errors in X (left-right direction), Y (head-foot direction), and Z (abdomen-back direction) directions of the ICTID arms-up, TAFID, ICTID arms-down and HNSID groups were (0.15 ± 0.18) cm, (0.15 ± 0.16) cm, (0.16 ± 0.16) cm, and (0.15 ± 0.20) cm; (0.15 ± 0.15) cm, (0.21 ± 0.25) cm, (0.28 ± 0.23) cm, and (0.27 ± 0.21) cm; (0.13 ± 0.14) cm, (0.15 ± 0.14) cm, (0.17 ± 0.13) cm, and (0.16 ± 0.14) cm, respectively. Among them, the ICTID arms-up group had the minimal setup errors in X direction than those in ICTID arms-down (p=0.001) and HNSID groups (p=0.001), and in Y direction than those in TAFID (p<0.001), and in Z direction than those in ICTID arms-down (p<0.001) and TAFID groups (p=0.034). For the rotational errors of the four groups in the directions of sagittal plane, transverse plane, and coronal plane, the ICTID arms-up group had the smallest setup errors in the sagittal plane than that of TAFID groups and similar rotation setup errors with those of the other three groups.

**Conclusion:**

For patients requiring radiation of primary lung lesion and positive supraclavicular lymph nodes, an integrated frame fixation device is preferred the ICTID arms-up methods provide the smallest set up error and satisfied repeatability of body position, compared with TAFID and HNSID.

## 1 Introduction

Lung cancer is the leading cancer-related death cause worldwide (1). Supraclavicular lymph node metastasis is not rare in patients with locally advanced lung cancer; it is present in 12% of patients at the time of their first diagnosis and in up to 37.5% of autopsy cases (2). Previous studies have shown that local radiotherapy involving the supraclavicular area has a curative effect on patients with metastasis in this area (3–5). Ensuring high setup accuracy of this area during radiotherapy critical.

A thoracoabdominal flat immobilization device (TAFID) suitable for radiotherapy in most patients with lung cancer (6). However, owing to the wide range of motion in the acromioclavicular joint, the setup repeatability of the supraclavicular area is unsatisfactory. Therefore, improvements in the setup repeatability are worth exploring. Our previous studies have compared different immobilization methods for patients with thoracic tumors, such as esophageal and lung cancer (7–9), and found that the overall setup accuracy when using a TAFID is less reliable than integrated cervicothoracic immobilization devices (ICTID) (7–9). In other hands, head-neck-shoulder immobilization device (HNSID) is used in patients with tumor located in upper lobe of lungs, as the motion of lungs below the tracheal carina have little influence on the setup error of target volume. Consequently, the current study first evaluated the differences between TAFID, ICTID and HNSID, in terms of overall setup error and stability of the supraclavicular region for lung cancer patients.

## 2 Materials and methods

### 2.1 Patients

We prospectively enrolled 100 patients with lung cancer who received thoracic radiotherapy at our center from October 2019 to September 2022. The inclusion criteria were as follows: (1) The radiotherapy target area included the supraclavicular area. (2) The patient underwent conventional fractionated radiotherapy. (3) The patient underwent cone-beam computer tomography (CBCT) check five times in the first week and one time per week during the radiotherapy. (4) The Karnofsky score was greater than 70.

The study was approved by the ethics review boards of our institution, and all patients were provided with signed informed consent prior to enrolment.

### 2.2 Patient position immobilization and CT simulation

**Figure 1:**
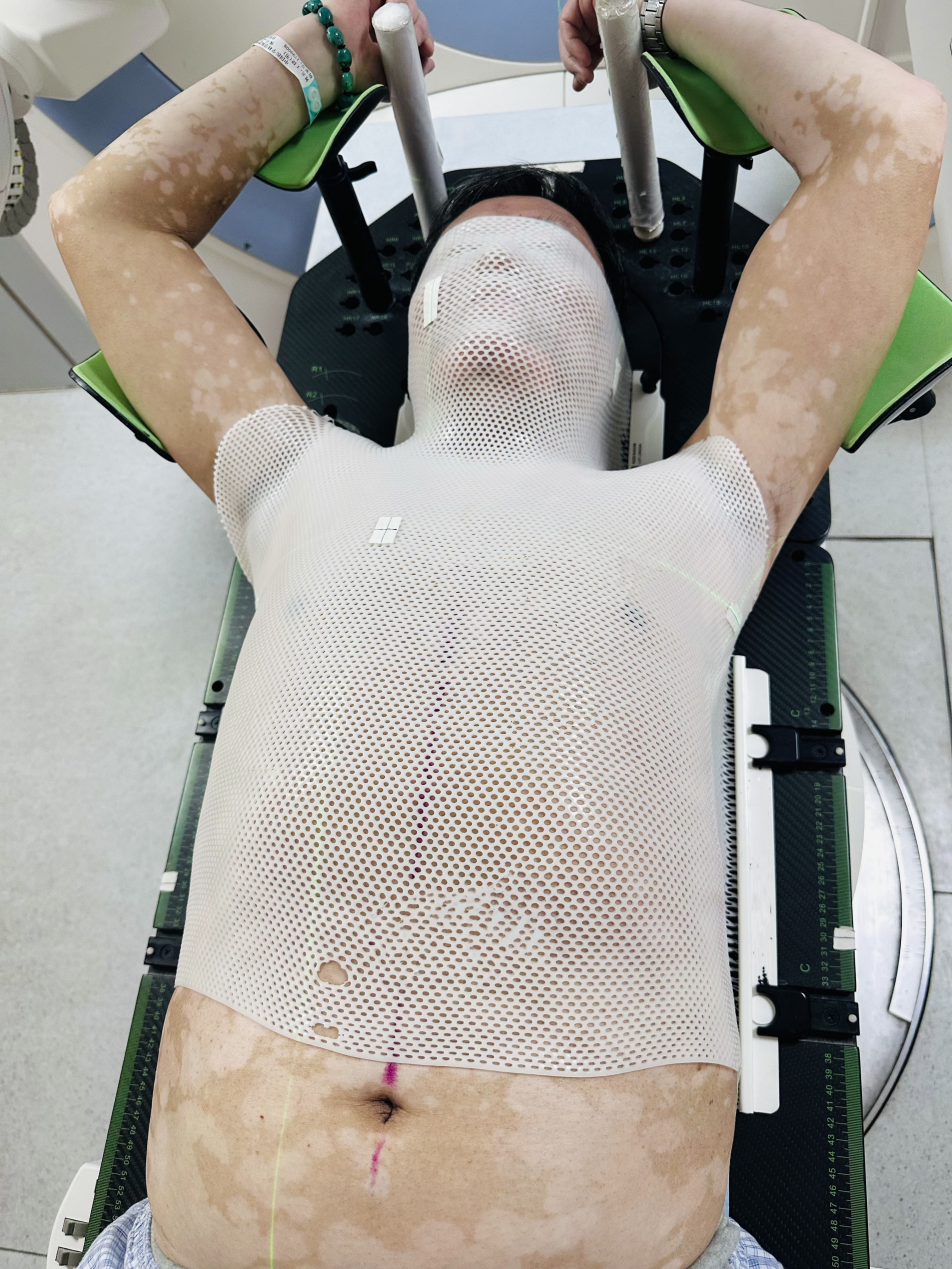
Illustration of the setup using the integrated cervicothoracic immobilization devices (ICTID) with arms on brackets.

**Figure 2:**
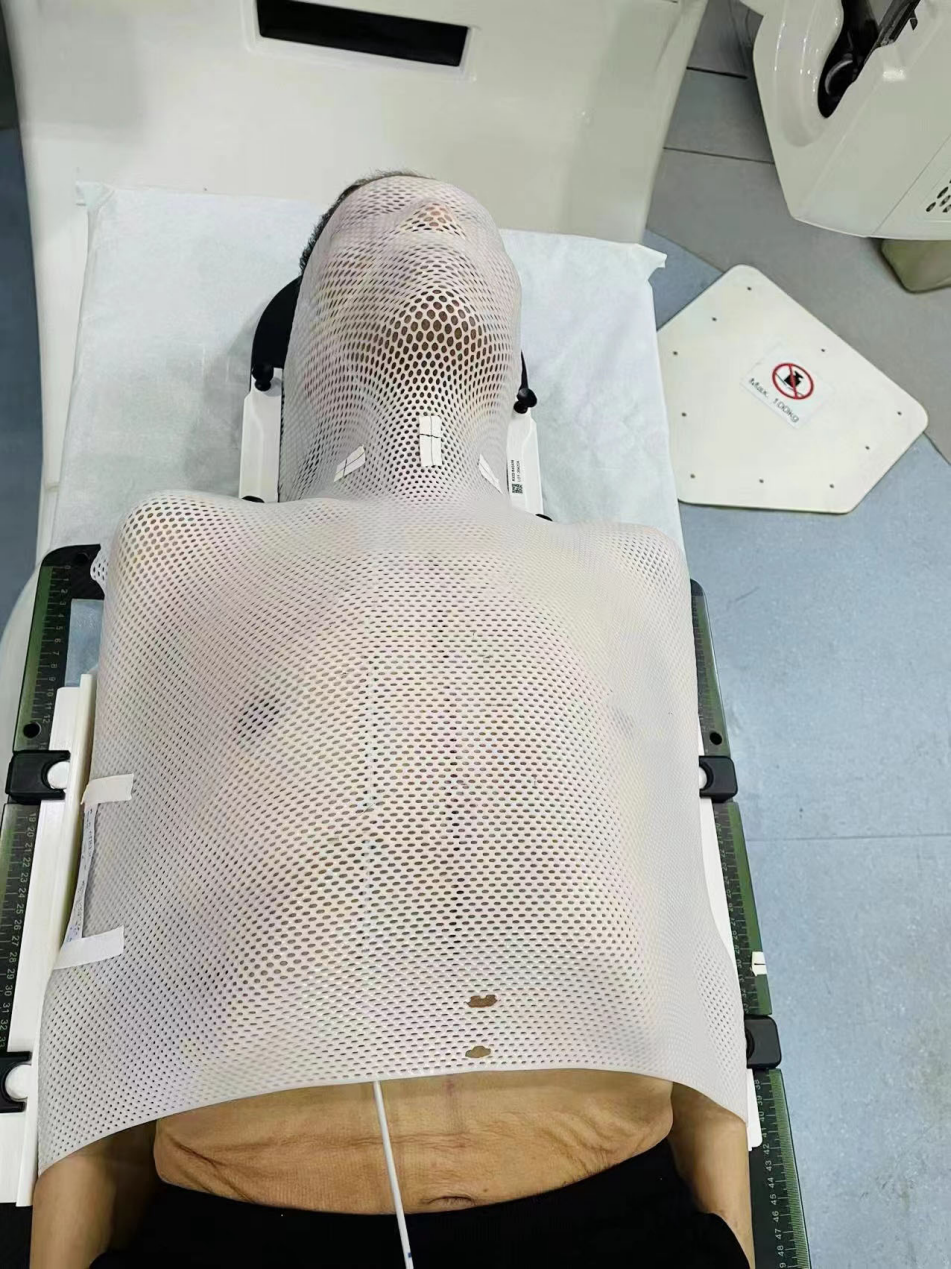
Illustration of the setup using the integrated cervicothoracic immobilization devices (ICTID) with arms on body sides.

**Figure 3:**
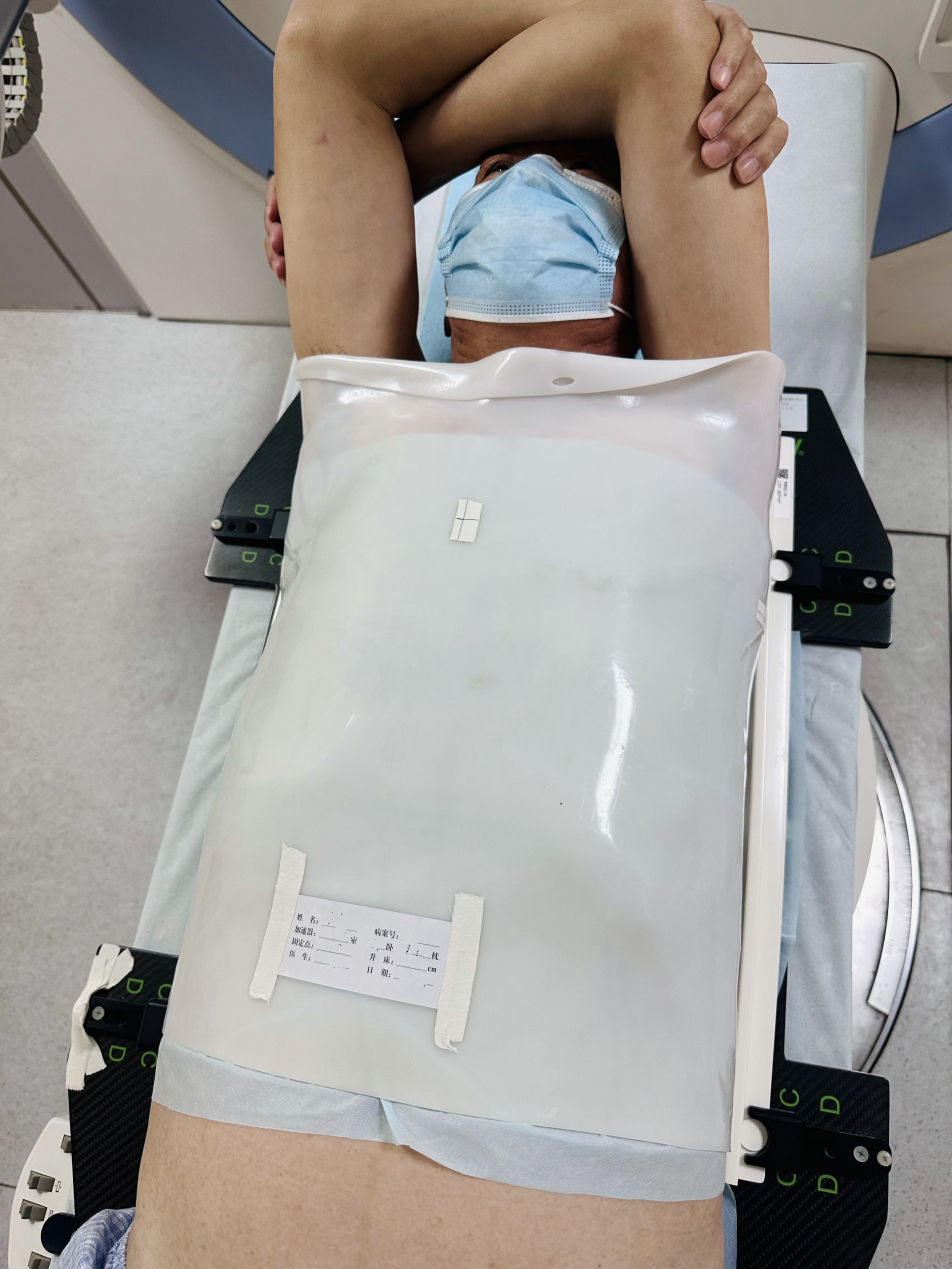
Illustration of the setup using the thoracoabdominal flat immobilization device (TAFID).

**Figure 4:**
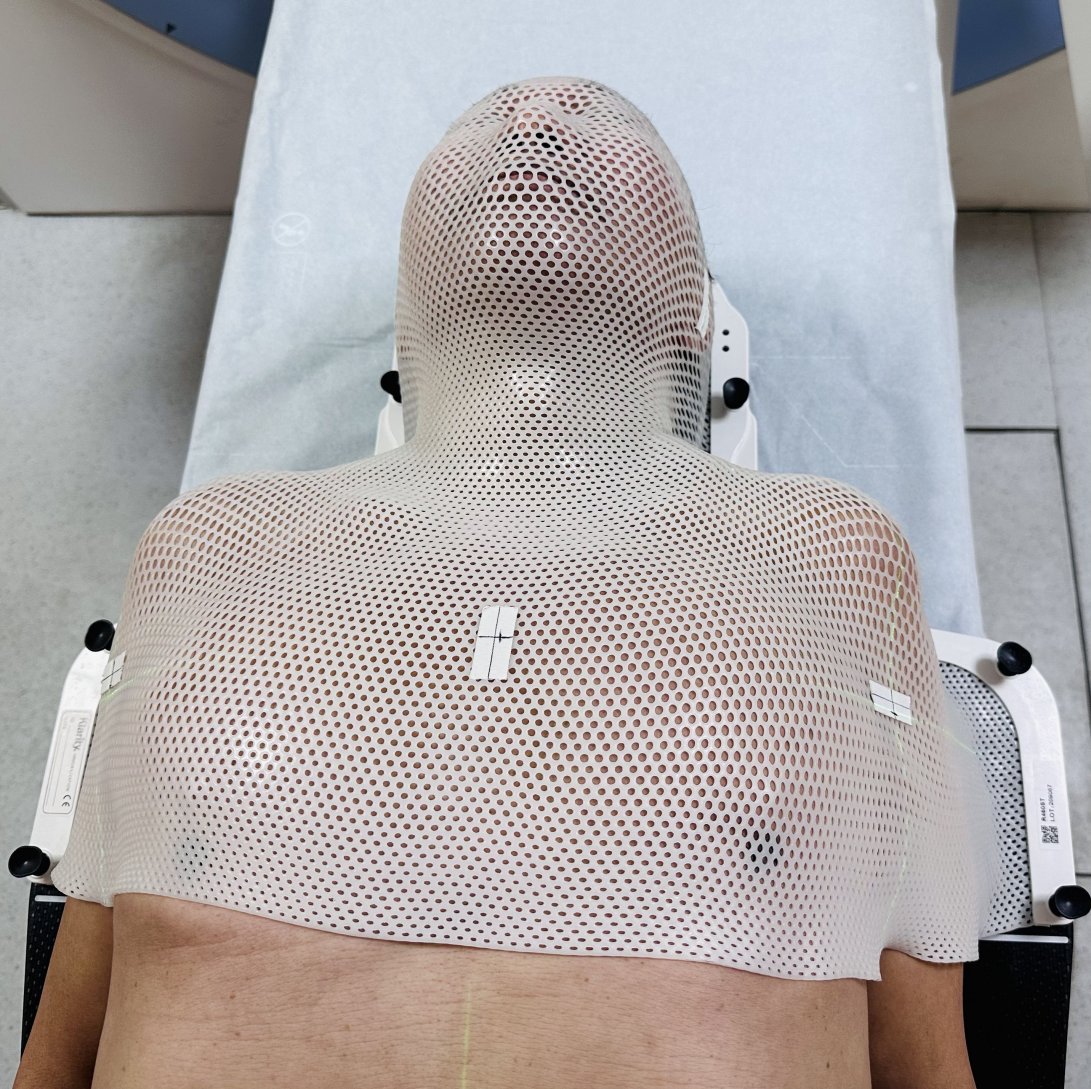
Illustration of the setup using the head-neck-shoulder immobilization device (HNSID).

Details of simulation, target volume definition, prescription, planning were published previously (10). The clinical target volume (CTV) was created by expanding the gross tumor volume 0.6–0.8 cm, as well as ipsilateral hilum, mediastinal and supraclavicular nodal stations involved. The planning target volume (PTV) was generated by a uniform 0.5 cm expansion around the CTV, which is enough to cover 95% setup errors on the basis of our institutional data, regardless of definitive or adjuvant radiotherapy for lung cancer (10–12). Patients in the supine position were fixed using four different position immobilization methods and the corresponding films in a calm environment. In the ICTID arms-up group, patients were required to have the upper part of their body well exposed and arms naturally extended approximately 120°, while arms and wrists were placed on brackets, holding a stanchion in their hands (**Figure 1**). In the ICTID arms-down group, both arms were placed on both sides and fixed with thermoplastic film (**Figure 2**). In the TAFID group, the arms were crossed in front of the forehead, and the thermoplastic membrane was fixed (**Figure 3**). In the HNSID group, the patient’s arms were placed on both sides of the body in a relaxed state, and the thermoplastic membrane was fixed (**Figure 4**). The angle and height of the arm bracket and head restraint model were adjusted according to the patient’s comfort and clinical needs to ensure setup repeatability in the ICTID arms-up group. CT simulation scanning (Philips Brilliance Big Bore or Siemens SOMATOM D Definition AS 40) was performed under free breathing conditions. The positioning center should be as close as possible to the sternoclavicular joint.

### 2.3 Target area delineation and plan design

Patient positioning images were uploaded to the Pinnacle (version 9.10) treatment planning system. The doctor drew the target area based on the principles of the field involved. Physicians follow the ICRU No. 83 report to formulate treatment plans. All patients were treated with intensity-modulated radiotherapy (including static intensity-modulated therapy, volumetric modulated arc therapy, and integrated intensity-modulated therapy). If the VMAT was selected, the virtual block structure was created to block the beamlets if necessary by closing the multi-leaf collimator (MLC) at prerequisite angles. In our study, the virtual block structure for arms protection was shown in violet and contoured based on the algorithm (13). The optimization parameters for the virtual block structure: Dmax<5 Gy and the primary optimization weight for the block was initially set to 10 and could be adjusted to 20 (maximum weight value) step-by-step according to the composite object values in the Pinnacle treatment planning system.

### 2.4 Image guidance and data acquisition

During the radiotherapy setup, patient placement was based on the surface marking line along with the positioning line on the thermoplastic film and laser guidance. CBCT scanning was performed for position calibration on a daily basis in the first five radiotherapy fractions, and then once a week until the last time. In CBCT images, the upper boundary of the scan should not exceed the cricoid cartilage, while the lower boundary should not exceed 5 cm below the septum muscle or 20 cm above and below the treatment center. The anterior boundary includes subcutaneous soft tissue, while the posterior boundary includes the entire vertebral body. According to the ICRU recommendations, the scope of the registration frame should be 2 cm away from the PTV in three dimensions. CBCT image registration was performed based on soft tissue window registration. The target area was used as a reference to manually fine-tune and record the setup errors for the six degrees of freedom, namely, the overall translation errors in the X (left-right direction), Y (head-foot direction), and Z (ventral-dorsal direction) directions, and rotational errors in Rx (sagittal plane), Ry (transverse plane), and Rz (coronal plane). Correction and adjustment are made for the surface marking line along with the positioning line on the thermoplastic film, based on setup error data of the first 5 CBCT. In the following fractions, we verified the setup by CBCT once a week. If the setup error is more than 5 mm or 2°, more CBCT will be request until the setup error is acceptable.

The sternoclavicular joint displacements, namely, Xst, Yst, and Zst, were obtained considering the posterior cervical spine as the region of interest and using manual registration. The acromioclavicular joint displacements, namely, Xac, Yac, and Zac, were obtained by manual calibration in the CBCT images of the region of interest.

### 2.5 Definition of displacement error

Systematic error Σ (standard deviation of average individual case error) and random error σ (root mean square of the standard deviation regarding the individual case error). The relative displacements of the upper and lower clavicles were calculated by ΔX =|X_st_ − X_ac_|, ΔY =|Y_st_ − Y_ac_|, and ΔZ =|Z_st_ − Z_ac_|. Therefore, the displacement amplitude was defined as:


$$d=\sqrt{\Delta X^{2}+\Delta Y^{2}+\Delta Z^{2}}$$

### 2.6 Statistical analysis

According to our previous retrospective study (7), 25 patients were needed for each groups at least. All patients were randomized by random number tables (14). SPSS25.0 software was used to compare the overall setup error, sternoclavicular joint setup error, acromioclavicular joint setup error, and displacement amplitude of the sternoclavicular joint against the acromioclavicular joint using t-test or rank sum test (with a significance level of 0.05).

## 3 Results

### 3.1 Characteristics of patients

Our patient population was predominantly male (79% men *vs*. 21% women), with a median age of 62 years. Half of patients presented with large tumors or involved mediastinum (T3-4 51.0%). The primary tumours were more located in the right lung (58%). An average of 8.3 sets of CBCT was done for per patients (**Table 1**).

**Table 1:** Baseline characteristics of patients.

	ICTID arms-up (n=25)	TAFID (n=25)	ICTID arms-down (n=25)	HNSID (n=25)	F score	P value
Age (median, range, yrs)	62 (46-71)	63 (40-75)	62 (30-82)	64 (50-77)	1.500	0.220
BMI					1.588	0.197
<24	13	9	15	12		
≥24	12	16	11	13		
Gender					0.371	0.774
Male	21	18	20	20		
Female	4	7	5	5		
KPS					1.741	0.164
80	14	18	21	21		
90	11	7	4	4		
Primary lesion					0.432	0.730
Left lung	12	11	8	11		
Right lung	13	14	17	14		
T Stage					0.247	0.863
I	4	5	2	2		
II	9	10	9	8		
III	7	3	7	4		
IV	5	7	7	11		
CBCT sets	195	206	209	225	1.703	0.172

BMI, body mass index;

CBCT, cone-beam computer tomography;

HNSID, head-neck-shoulder immobilization device;

ICTID, integrated cervicothoracic immobilization devices;

KPS, Karnofsky score;

TAFID, thoracoabdominal flat immobilization device.

### 3.2 Setup error and distribution of the four immobilization methods

A total data of 825 sets of CBCT were obtained. The overall target positioning errors in the four immobilization methods are listed in **Table 2**. The translation error values of the ICTID arms-up group in the head-foot direction, ventral-dorsal direction, and Rx (sagittal plane) were smaller than those of the other three groups. In X (left-right direction), there was a significant difference in the ICTID arms-up group compared with the ICTID arms-down and HNSID groups (P<0.05). In Y (head-foot direction), there was a significant difference between the ICTID arms-up and TAFID groups (P<0.05). In Z (ventral and dorsal direction), there was a significant difference in the ICTID arms-up group compared with the ICTID arms-down and TAFID groups (P<0.05). Regarding the rotation error, there was a significant difference in the sagittal plane direction between the ICTID arm-up and TAFID groups (P<0.05).

**Table 2:** Overall setup errors for the four immobilization methods.

	ICTID arms-up (A) (n=25)	TAFID (B) (n=25)	ICTID arms-down (C) (n=25)	HNSID arm-side (D) (n=25)	A *vs*. B		A *vs*. C		A *vs*. D	
					T	P	T	P	T	P
ΔX (cm, mean ± SD)	0.15 ± 0.18	0.15 ± 0.16	0.16 ± 0.16	0.15 ± 0.20	0.069	0.945	3.304	0.001	3.318	0.001
ΔY (cm, mean ± SD)	0.15 ± 0.15	0.21 ± 0.25	0.28 ± 0.23	0.27 ± 0.21	-4.782	0.000	-0.620	0.535	1.284	0.200
ΔZ (cm, mean ± SD)	0.13 ± 0.14	0.15 ± 0.14	0.17 ± 0.13	0.16 ± 0.14	2.126	0.034	5.020	0.000	0.374	0.708
ΔRx (°, mean ± SD)	0.78 ± 0.48	0.83 ± 0.56	0.92 ± 0.41	0.81 ± 0.54	4.737	0.000	-1.544	0.123	1.175	0.240
ΔRy (°, mean ± SD)	0.76 ± 0.57	0.72 ± 0.65	1.00 ± 0.62	0.98 ± 0.81	-0.112	0.911	1.096	0.274	-0.385	0.700
ΔRz (°, mean ± SD)	0.76 ± 0.64	0.72 ± 0.59	0.86 ± 0.56	0.78 ± 0.77	-1.398	0.164	-0.900	0.368	1.803	0.072

Δ, change;

HNSID, head-neck-shoulder immobilization device;

ICTID, integrated cervicothoracic immobilization devices;

TAFID, thoracoabdominal flat immobilization device;

SD, Standard deviation;

T> 4.303 or < -4.303 is considered statistically significant;

P<0.05 is considered statistically significant.

The translation setup error distributions in the X, Y, and Z directions, and the rotation errors in the sagittal, transverse, and coronal planes of the four groups are reported in **Table 3**. The percentages of X ≤ 2 mm in the ICTID arms-up, TAFID, ICTID arms-down, and HNSID groups were 57%, 57%, 67%, and 56%, respectively, 47%, 42%, 39%, and 43%, respectively, in Y, and 68%, 68%, 65%, and 64%, respectively, in Z (ventral-dorsal direction). The percentages of rotation error ≤ 1° are 73%, 57%, 80%, and 72% in Rx; 72%, 72%, 69%, and 60% in Ry; 69%, 71%, 72%, and 69% in Rz, respectively.

**Table 3:** Setup error distribution of the four immobilization methods.

	ICTID arms-up (n=25)			TAFID (n=25)			ICTID arms-down (n=25)			HNSID (n=25)		
	>4mm	≤4mm and >2mm	≤2mm	>4mm	≤4mm and >2mm	≤2mm	>4mm	≤4mm and >2mm	≤2mm	>4mm	≤4mm and >2mm	≤2mm
ΔX (mm)	8.0%	35.0%	57.0%	5.0%	38.0%	57.0%	9.0%	24.0%	67.0%	10.0%	34.0%	56.0%
ΔY (mm)	4.0%	49.0%	47.0%	17.0%	41.0%	42.0%	28.0%	33.0%	39.0%	30.0%	27.0%	43.0%
ΔZ (mm)	2.0%	30.0%	68.0%	6.0%	26.0%	68.0%	5.0%	30.0%	65.0%	8.0%	28.0%	64.0%
	ICTID arms-up (n=25)			TAFID (n=25)			ICTID arms-down (n=25)			HNSID (n=25)		
	>2°	≤2°and >1°	≤1°	>2°	≤2°and >1°	≤1°	>2°	≤2°and >1°	≤1°	>2°	≤2°and >1°	≤1°
ΔRx (°)	3.0%	24.0%	73.0%	4.0%	39.0%	57.0%	5.0%	31.0%	80.0%	4.0%	24.0%	72.0%
ΔRy (°)	3.0%	25.0%	72.0%	3.0%	25.0%	72.0%	4.0%	27.0%	69.0%	13.0%	27.0%	60.0%
ΔRz (°)	4.0%	27.0%	69.0%	4.0%	25.0%	71.0%	6.0%	22.0%	72.0%	8.0%	23.0%	69.0%

Δ, change;

HNSID, head-neck-shoulder immobilization device;

ICTID, integrated cervicothoracic immobilization devices;

TAFID, thoracoabdominal flat immobilization device.

### 3.3 Three-dimensional displacement and displacement amplitude of acromioclavicular joint

**Table 4** illustrates that the three-dimensional acromioclavicular joint displacement and displacement amplitude of the acromioclavicular joint in the ICTID arms-up group were smaller than those in the other three groups. There was a significant difference in the displacement direction of the acromioclavicular joint among the three groups (P<0.05).

**Table 4:** Displacement of the acromioclavicular joint of the four immobilization methods.

	ICTID arms-up (A)	TAFID (B)	ICTID arms-down (C)	HNSID arm-side (D)	A *vs*. B		A *vs*. C		A *vs*. D	
					Z	P	Z	P	Z	P
ΔX (cm)	0.09 ± 0.10	0.10 ± 0.12	0.13 ± 0.10	0.12 ± 0.10	-0.901	0.368	-5.383	0.000	-4.199	0.000
ΔY (cm)	0.14 ± 0.13	0.13 ± 0.13	0.18 ± 0.14	0.16 ± 0.13	-0.77	0.939	-4.330	0.000	-1.767	0.077
ΔZ (cm)	0.12± 0.11	0.16 ± 0.15	0.17± 0.16	0.11± 0.11	-2.729	0.006	-3.622	0.000	-0.459	0.646
d (cm)	0.24± 0.15	0.27 ± 0.18	0.32± 0.18	0.26± 0.14	-2.157	0.031	-5.532	0.000	-2.746	0.006

HNSID, head-neck-shoulder immobilization device;

ICTID, integrated cervicothoracic immobilization devices;

TAFID, thoracoabdominal flat immobilization device.

Z < -1.96 is considered statistically significant;

P<0.05 is considered statistically significant.

## 4 Discussion

Patients with lung cancer due to complex causes require personalized treatment. For patients administered with radiotherapy, including the mediastinum, supraclavicular area, and primary lung lesion, clinicians in charge have different opinions on the method of body position immobilization. In the past, TAFID and HNSID were used to fix the body position during intensity-modulated radiotherapy for lung cancer (15). However, due to the lack of positional restrictions, such as with TAFID on the neck and HNSID in the lower mediastinum area, setup errors between different fractions in patients who need treatment in the supraclavicular and lower mediastinum areas are unacceptable. The unconscious autonomous movements of organ also result in large setup errors and poor setup repeatability. Compared with the arm-head-hugging posture, patients holding the arm-lifting posture have more accuracy in shoulder blades, more relaxation in the neck and back, fit the bed surface more closely, and more repeatability of the arm position, which overcomes the instability caused by the patient’s “shrug” or “droop” action. Therefore, the repeatability of the ventral-dorsal direction improved. Consequently, for patients whose treatment target area includes the supraclavicular area, a new method that can consider the advantages of the two types of body frames is needed to control the patients’ unconscious autonomous movements to reduce positioning error. In this study, we found that the overall setup error was significantly reduced when ICTID was used along with the arm lift posture. In particular, by comparing the displacements of the acromioclavicular and sternoclavicular joints between different fractions, we concluded that the ICTID arms-up positioning had advantages over flat immobilization devices in fixing the supraclavicular area.

Interfraction setup errors in lung cancer radiotherapy may affect the dose distribution in the target area and endanger normal tissues. Small setup error will maintain the minimal PTV margins necessary for SBRT and, consequently, reduce normal tissue complication probability and increase tumor control probability (16–20). Roper et al. showed no significant loss of target volume coverage when a plan isocenter rotational error of 0.5°was simulated, although rotational errors up to 2°resulted in significant loss of target coverage (19). Therefore, it is necessary to minimize the error to improve radiotherapy accuracy. Our results demonstrate that the errors in the Y, Z, and Rx directions of the ICTID arms-up group are significantly smaller than those of the TAFID group, which is closely related to the larger fixation area of the ICTID. Using the ICTID, compared with the TAFID, the neck and head of the patient can be better fixed so that the midline of the patient can coincide with the top laser. This ensures better repeatability of the patient’s position and reduce the setup error in the head-foot direction. Moreover, because the ICTID has wrist and arm support, it can achieve patient arm lift in a relatively comfortable and fixed position, reducing the degree of traction caused by the improper placement of the arm lift and discomfort, which causes the ICTID arms-up group to exhibit smaller errors in the Z direction and Rx. Compared with the HNSID arm side group, the ICTID arms-up group had a significantly better immobilization effect on the chest and abdomen of patients, leading to a significant reduction in the left-right deflection of the patient’s midline and a significantly smaller error in the X direction. In summary, ICTID exhibited a better immobilization effect than the other two methods.

Lymphatic metastasis is one of the main causes of lung cancer metastasis. Cancer cells metastasize remotely through blood vessels around the lymph nodes or efferent lymphatic vessels (21). It is important to explore the relationship between accurate radiotherapy provided to lymph nodes in the drainage area of metastatic lymph nodes and the overall survival of patients. We focused on displacement in the supraclavicular and infraclavicular regions in patients with lung cancer, which is related to increased mortality. The relative displacements of the sternoclavicular and acromioclavicular joints correspond to the amplitude of the shoulder joint movement compared to the simulation position. The movement of the acromioclavicular joint, lifting height and abduction range of the upper and lower arms, and rotation of the pillow pose are the requirements for setup repeatability. The ICRU No. 24 report suggested that a 5% deviation in the dose change in the target area would increase the recurrence of the primary focus and complications related to endangering organs. The ICTID can adjust the angle and height of the arm and wrist supports so that the patient’s arms rest more relaxed and comfortably, further ensuring that both arms are far away from the supraclavicular area simultaneously with high repeatability to avoid unnecessary radiation. The adjustment of the position and model of the pillow improves the consistency and stability of the rotation and pitch of the head and neck of the patient. Moreover, this reduces the possibility of injury to important organs, such as the spinal cord, thyroid, and brachial plexus. According to the statistical analysis conducted in this study, compared with the TAFID, ICTID arms-down, and HNSID groups, the ICTID arms-up group exhibited the smallest setup errors in terms of shoulder joint mobility, which greatly reduced the uneven dose distribution caused by the shoulder joint mobility changes. However, two points need to be clarified for implying the ICTID. First, the ICTID arms-down position is the best choice for the patients with disorder of shoulder joint, who can’t lift the arms up. Second, the treatment time including positioning, CBCT and dose delivery should not be too long, usually less than 10-20 minutes. Because one usually can’t maintain arms stable and immobile when lifting the arms above over 20 minutes.

Although the setup error is smaller using the ICTID arms-up method, the influence of the respiratory movement cannot be ignored. Research shows that free breathing can lead to artifacts and changes in the shape, size, density, and position of anatomical structures. The volume and spatial position deformation could not be predicted. Respiratory movement leads to uncertainty in dose delivery, which in turn leads to uncertainty in target coverage. The respiratory movement of patients can be reduced or eliminated through the deep inspiration breath hold (DIBH) technology to improve the accuracy of radiotherapy. Previous studies have proved that surface-guided radiation therapy (SGRT) technology can reduce the setup error in the supraclavicular region, reduce the uncertainty in the position between fractions, and monitor the patient movement in the fractions during treatment. However, this technology also presents the characteristics of expensive equipment, the heavy economic burden on patients, and the need to train patients before treatment, which makes the operation complex. Abdominal compression combined with SGRT can effectively reduce tumor motion and target volume.

The limitations of this study are as follows. First, the study was conducted at a single center. Second, the study did not refine the location(upper, middle or lower lobe) and the number of primary lung lesions, which were not balanced among the four groups. For the primary lung lesions located in the lower lobe, it is more difficult to achieve good setup for lung lesion and supraclavicular station at the same time, compared with the ones with lung lesion located in the upper lobe. Third, we did not apply CBCT before every fraction as the CBCT was not covered be medical assurance during the study period. In regards of cost-effectiveness, around 30-40% treatment fractions were guided with CBCT (6). Thus parts of interfraction setup errors were not obtained, which had negative impact on the quality of our study. At last, the quality of image registration during the clinical treatment process was not controlled systematically. In future research, the experimental conditions should be optimized and gradually extended to dosimetric comparisons to provide a more accurate clinical setting reference.

In summary, radiotherapy for lung cancer is a multigroup collaborative process. Cancer patients need to be administered personalized treatments according to their conditions. Irradiation of the tumor target area and lymphatic drainage area during postural immobilization is particularly important. The change in tumor location during the treatment of lung cancer poses severe challenges to the treatment (22, 23). This study has reference significance for body position immobilization in lung cancer radiotherapy. For lung cancer patients with supraclavicular target radiotherapy, the setup error of the ICTID arms-up method is smaller, which can better reduce the external expansion of the PTV, providing a better guarantee for patients. The ICTID arms-up and TAFID groups had smaller setup errors in the X and Z directions than that of the ICTID arms-down group. Simultaneously, lifting the arms with hands holding the poles can reduce unnecessary radiation to the arms. Therefore, when lung cancer requires a chest clavicle combined with mediastinal radiotherapy, the ICTID arms-up immobilization method is recommended.

## Data availability statement

The raw data supporting the conclusions of this article will be made available by the authors, without undue reservation.

## Ethics statement

The studies involving human participants were reviewed and approved by National GCP Center for Anticancer Drugs, The independent Ethics Committee. The patients/participants provided their written informed consent to participate in this study.

## Author contributions

BW and SL collected data, analyze data, interpreted the study results, and drafted the manuscript. XF, WQ, HS, LH, KZ, SW, ZZ, ZX, DC, QF, XW and FH collected data, analyze data, revised manuscript critically for important intellectual content. JW designed and supervised the study, and revising manuscript critically for important intellectual content. All authors contributed to the article and approved the submitted version.
