# Corrigendum: Superiority of integrated cervicothoracic immobilization in the setup of lung cancer patients treated with supraclavicular station irradiation

**DOI:** 10.3389/fonc.2023.1276782

**Published:** 2023-08-24

**Authors:** Bao Wan, Shihong Luo, Xin Feng, Wenhua Qin, Haifan Sun, Lu Hou, Kun Zhang, Shiyu Wu Zongmei Zhou, Zefen Xiao, Dongfu Chen, Qinfu Feng, Xin Wang, Fukui Huan, Nan Bi, Jianyang Wang

**Affiliations:** Department of Radiation Oncology, National Cancer Center/National Clinical Research Center for Cancer/Cancer Hospital, Chinese Academy of Medical Sciences and Peking Union Medical College, Beijing, China

**Keywords:** lung cancer, radiotherapy, positioning error, thoracoabdominal flat immobilization device, integrated cervicothoracic immobilization device

In the published article, there was an error in **Figure 1** as published. The image contains PRIVATE INFORMATION of the patient. The correct **Figure 1** appear below.

**Figure 1:**
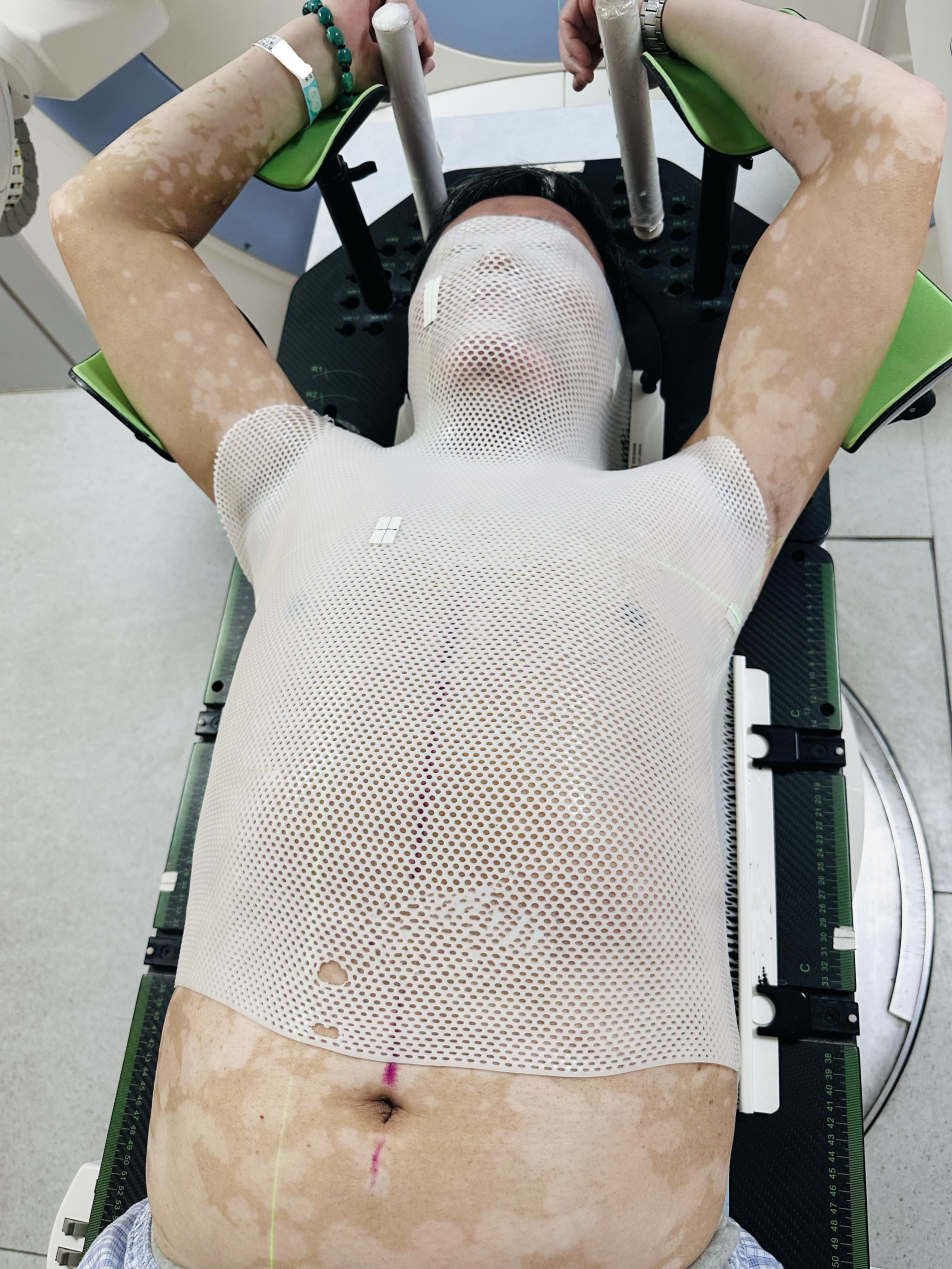
Illustration of the setup using the integrated cervicothoracic immobilization devices (ICTID) with arms on brackets.

The authors apologize for this error and state that this does not change the scientific conclusions of the article in any way. The original article has been updated.

